# Discovery and clinical introduction of first-in-class imipridone ONC201

**DOI:** 10.18632/oncotarget.11814

**Published:** 2016-09-01

**Authors:** Joshua E. Allen, C. Leah B. Kline, Varun V. Prabhu, Jessica Wagner, Jo Ishizawa, Neel Madhukar, Avital Lev, Marie Baumeister, Lanlan Zhou, Amriti Lulla, Martin Stogniew, Lee Schalop, Cyril Benes, Howard L. Kaufman, Richard S. Pottorf, B. Rao Nallaganchu, Gary L. Olson, Fahd Al-Mulla, Madeleine Duvic, Gen Sheng Wu, David T. Dicker, Mala K. Talekar, Bora Lim, Olivier Elemento, Wolfgang Oster, Joseph Bertino, Keith Flaherty, Michael L. Wang, Gautam Borthakur, Michael Andreeff, Mark Stein, Wafik S. El-Deiry

**Affiliations:** ^1^ Oncoceutics, Inc., Philadelphia, PA, USA; ^2^ Fox Chase Cancer Center, Philadelphia, PA, USA; ^3^ University of Texas MD Anderson Cancer Center, Houston, TX, USA; ^4^ Weill Cornell Medicine, New York, NY, USA; ^5^ Massachusetts General Hospital, Boston, MA, USA; ^6^ Harvard Medical School, Boston, MA, USA; ^7^ Rutgers Cancer Institute of New Jersey, New Brunswick, NJ, USA; ^8^ Provid Pharmaceuticals, Monmouth Junction, NJ, USA; ^9^ Kuwait University Medical School, Kuwait; ^10^ Karmanos Cancer Institute, Detroit, MI, USA; ^11^ The Children's Hospital of Philadelphia, Philadelphia, PA, USA

**Keywords:** ONC201, TIC10, integrated stress response, ATF4, DRD2

## Abstract

ONC201 is the founding member of a novel class of anti-cancer compounds called imipridones that is currently in Phase II clinical trials in multiple advanced cancers. Since the discovery of ONC201 as a p53-independent inducer of TRAIL gene transcription, preclinical studies have determined that ONC201 has anti-proliferative and pro-apoptotic effects against a broad range of tumor cells but not normal cells. The mechanism of action of ONC201 involves engagement of PERK-independent activation of the integrated stress response, leading to tumor upregulation of DR5 and dual Akt/ERK inactivation, and consequent Foxo3a activation leading to upregulation of the death ligand TRAIL. ONC201 is orally active with infrequent dosing in animals models, causes sustained pharmacodynamic effects, and is not genotoxic. The first-in-human clinical trial of ONC201 in advanced aggressive refractory solid tumors confirmed that ONC201 is exceptionally well-tolerated and established the recommended phase II dose of 625 mg administered orally every three weeks defined by drug exposure comparable to efficacious levels in preclinical models. Clinical trials are evaluating the single agent efficacy of ONC201 in multiple solid tumors and hematological malignancies and exploring alternative dosing regimens. In addition, chemical analogs that have shown promise in other oncology indications are in pre-clinical development. In summary, the imipridone family that comprises ONC201 and its chemical analogs represent a new class of anti-cancer therapy with a unique mechanism of action being translated in ongoing clinical trials.

## DISCOVERY OF ONC201

**Figure 1:**
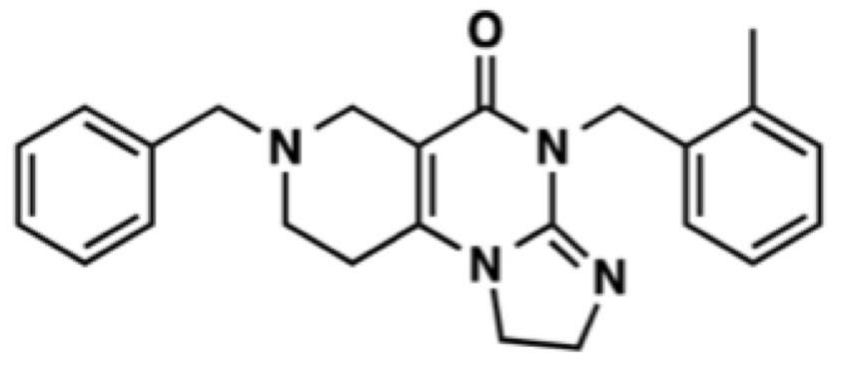
Molecular Structure of ONC201

ONC201 is a small molecule, chemical compound referred to as 7-benzyl-4-(2-methylbenzyl)-1,2,6,7,8,9-hexahydroimidazo [1,2-a]pyrido [3,4-e]pyrimidin-5(1H)-one that is the founding member of the imipridone class of compounds that share a unique heterocyclic pharmacophore (Figure 1). The compound was identified as a candidate anti-cancer lead compound in a luciferase reporter screen for small molecule p53-independent inducers of TNF-related apoptosis-inducing ligand (TRAIL) gene transcription in (TRAIL-resistant) bax-null HCT116 human colorectal cancer (CRC) cells [1, 2]. As a key effector of the immune-surveillance of cancer, TRAIL is a protein conditionally expressed on the surface of immune cells that triggers apoptosis in proximal tumor cells while not harming normal host cells [3]. Thus, the therapeutic concept that motivated the screen that identified ONC201 was to identify compounds that could safely upregulate a natural anti-tumor immune surveillance and cancer suppression mechanism with a desirable therapeutic index.

In the screen for TRAIL-inducing compounds, small molecules with pronounced capability to induce TRAIL and tumor cell death included ONC201 and the ER stress-inducing compound breflate, the pro-drug of Brefeldin A [2]. Interestingly, both ONC201 and breflate induced TRAIL at 36 to 48 hours post-treatment in tumor cells, suggesting that this phenomenon may be a late-stage downstream signaling/effector mechanism. Comparative studies of the two small molecules revealed that both compounds are active *in vivo* and result in similar downstream signaling that includes dual Akt and ERK inactivation. However, ONC201 showed high selectivity of induction of cell death in malignant cells, unlike breflate that was toxic to normal cells [2]. This early observation suggested a partial, but not complete overlap, in mechanism of action between ONC201 and ER stress-inducing compounds that would be elucidated subsequently. Referred to as TRAIL-inducing compound 10 (TIC10) based on the phenotype underpinning its discovery as an anti-tumor agent, ONC201 was selected as the lead compound for clinical development due to its favorable therapeutic index, lack of genotoxicity, drug-like chemical properties, penetration of the blood-brain barrier, p53-independent efficacy in a panel of refractory solid tumor cell lines, and single-dose anti-tumor activity *in vivo* [1, 2].

## MECHANISM OF ACTION

**Figure 2:**
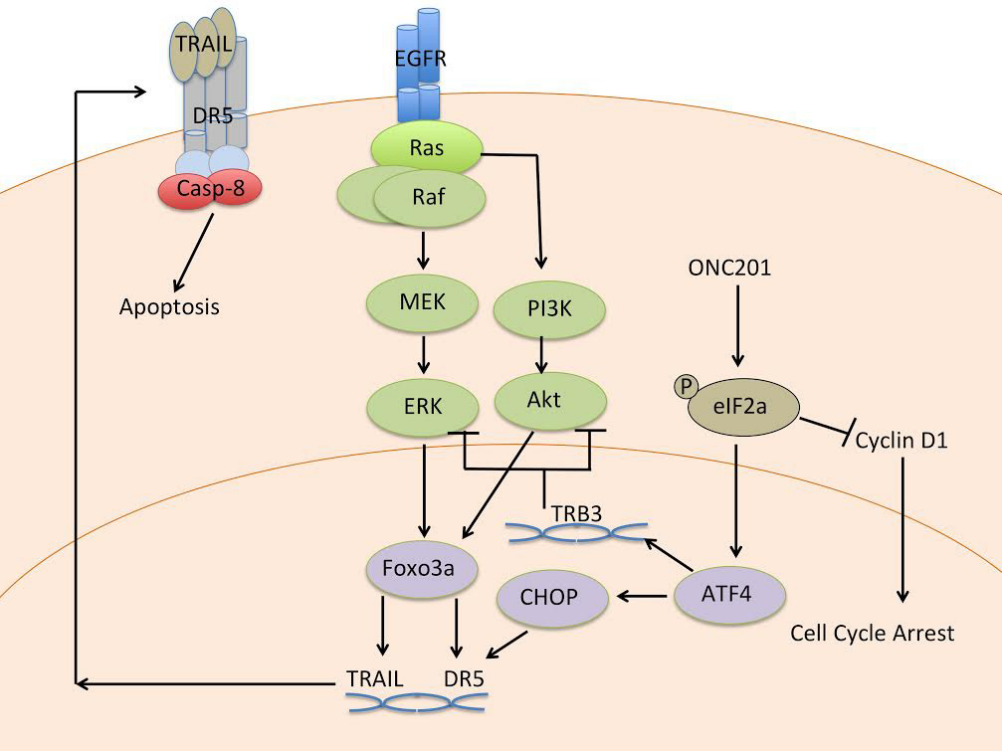
Mechanism of action of ONC201

The discovery of ONC201 by a phenotypic cell-based screen, rather than a target structure-based approach, meant that its precise mechanism of action (Figure 2) and direct molecular target was unknown at the time of discovery. The phenotypic screen, however, allowed for selection of a specific downstream molecular signaling pathway effect, anti-tumor effects that required the molecule to engage its target in a cellular context and trigger transcriptional events that also remained to be defined. Biochemical studies indicated that ONC201-mediated TRAIL upregulation was likely transcriptional based on observations of increased TRAIL mRNA levels in ONC201-treated cancer cells. Gene expression profiling (GEP) studies were performed in ONC201-sensitive p53-deficient HCT116 CRC cells at 48 hrs post-treatment to identify transcriptional changes coincident with TRAIL induction that could point to a common upstream regulator, e.g. transcription factor [1]. An in silico analysis of overlap between transcription factors with binding sites within the TRAIL gene promoter and potential transcriptional regulators of the mRNA changes observed in the GEP studies was performed. This cross-referencing suggested that Foxo3a, which possesses a binding site within the TRAIL gene promoter [4], could be activated in response to ONC201. Upregulation of Foxo3a target genes [5] by ONC201 was validated by RT-PCR and subcellular localization assays revealed that Foxo3a is indeed activated by ONC201 where it is translocated into the nucleus to transactivate the TRAIL gene. Chromatin-immunoprecipitation assays verified a dose-dependent increase in the amount of Foxo3a bound to the TRAIL gene promoter in response to ONC201. Both ONC201-induced TRAIL and overall anti-tumor efficacy was partially dependent on Foxo3a, as shown by RNA interference experiments in CRC models [1].

Evaluation of Foxo3a regulators in cell-based assays identified the pro-survival kinases Akt and ERK as synergistic drivers of ONC201-induced Foxo3a activation and translocation to the nucleus [6]. Further characterization of this phenomenon revealed that ONC201 inactivated Akt and ERK indirectly at late time points post-treatment that preceded downstream TRAIL induction. Akt and ERK inactivation in response to ONC201 treatment resulted in decreased phosphorylation of their enzymatic target sites on Foxo3a, Ser253 and Ser294, respectively. Dephosphorylation of Foxo3a presumably permits its release from cytoplasmic 14-3-3 proteins that normally bind to these phosphorylated residues, effectively inactivating Foxo3a by cytoplasmic sequestration. Genetic and pharmacological experiments validated that Akt and ERK inactivation has a synergistic effect on activating Foxo3a, TRAIL, and tumor cell death [1].

To investigate upstream signaling that may drive the late apoptotic effects of ONC201, GEP time course studies were undertaken in CRC and, independently, in non-Hodgkin's lymphoma cell lines [7, 8]. The GEP results in HCT116 and RKO CRC cells revealed an 11-gene ER stress response signature that was upregulated in response to ONC201 treatment. When investigated prospectively, this same gene signature was upregulated in Jeko-1 human mantle cell lymphoma cells in a time-dependent manner in response to ONC201 as early as 12 hours post-treatment. Many of these consensus signature genes induced by ONC201 have been shown to be regulated by ATF4 [9] and/or possess binding sites for the ATF4 transcriptional target gene CHOP [10]. ATF4 upregulation generally promotes apoptosis, in part by regulating the expression of two pro-apoptotic proteins, CHOP and the CHOP-target gene DR5 [11, 12]. Both CHOP and DR5 expression increased in a time-dependent manner with exposure to ONC201 in the GEP studies. Thus, these observations consistently suggested that ATF4 is being activated in tumor cells in response to ONC201 treatment.

To further validate the mechanism of ONC201, RKO cells with acquired and stable resistance to ONC201 were generated from the ONC201-sensitive parental cells. ONC201 is unable to induce cell death, or reduce the cell viability, of these cells with acquired resistance. Accordingly, ONC201-induced signaling effects related to the ATF4-pathway and Akt/ERK/Foxo3a/TRAIL signaling axes were not observed in the resistant cells [8]. Notably, these ONC201-resistant cells still responded to proteasome inhibitors, suggesting that the mechanism of resistance resides upstream of ATF4 induction.

ER stress induces ATF4/CHOP through the integrated stress response (ISR), which evolved to trigger cell death in response to a range of cellular stresses that include sustained ER stress, iron deficiency, amino acid deprivation, or viral infection. ISR induces its effects by causing increased phosphorylation of eukaryotic initiation factor 2 (eIF2α), which is essential in translation and inactivated by this phosphorylation event. eIF2α phosphorylation results in a general attenuation of translation but upregulates translation of ATF4 selectively, which subsequently induces CHOP [10, 13, 14]. In accordance with activation of ISR, ONC201 induces phosphorylation of eIF2α, which leads to its anti-cancer downstream signaling effects. Interestingly, TRB3 is a negative regulator of Akt that is induced in the ISR [15, 16] and its induction was observed in ONC201 GEP experiments. TRB3 knockdown prevented ONC201-mediated Akt inactivation, providing a link between ONC201 ISR activation and previously observed Akt inactivation [8].

**Figure 3:**
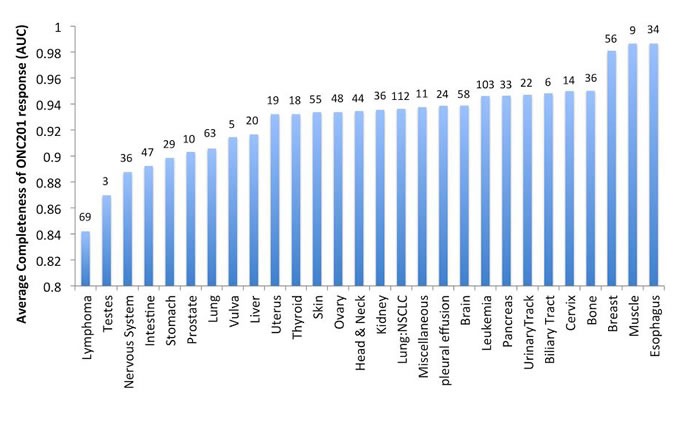
*In vitro* sensitivity of human cancer cell lines to ONC201 *In vitro* sensitivity of 1000 human cancer cell lines to ONC201 averaged and organized by tumor type. The results are shown as completeness of ONC201 response quantified as the area under the curve (AUC) in the dose-response cell viability curve averaged for all cell lines in each tumor type. Numbers above the bar indicate the number of cell lines tested per tumor type.

Further studies of how ONC201 causes eIF2α phosphorylation in the ISR revealed a mechanism that is independent of the ER stress sensor, PERK, and dependent on the double stranded RNA sensor PKR and HRI in solid tumor cells [8]. In lymphoma and leukemia cells, eIF2α phosphorylation was also PERK-independent but was dependent on GCN2 in the evaluated cell lines. Furthermore, ONC201-mediated ATF-4 induction resulted in inhibition of mammalian target of rapamycin complex (mTORC). Thus, ONC201 activates the ISR through a trigger that is distinct from proteasome inhibitors used to treat specific B-cell malignancies [7]. Consistent with the overlap of effector signaling pathways between ONC201 and proteasome inhibitors, lymphoma and multiple myeloma are most sensitive to ONC201 in preclinical *in vitro* studies with cell lines (Figure 3). Furthermore, ONC201-induced cell death or growth arrest has been associated with attenuation of XIAP and cyclin D1 expression, which is a diagnostic feature of mantle cell lymphoma [8].

Direct cell-free experiments suggest that ONC201 does not directly target kinases, epigenetic modulators, proteases, bromodomains, phosphodiesterases, or targets that have been previously associated with activation of ISR such as the proteasome, proteases, HSP70, HSP90, or deubiquitinases. Chemical affinity purification approaches have failed to generate a reproducible binding target candidate for ONC201, although RNA-binding proteins have been inconsistently detected. Thus, ONC201 does not engage many of the known cancer drug target mechanisms but clearly activates the ISR through a trigger that is distinct from available therapies that impact this same pathway.

A series of predictive, *in silico*, *in vitro*, and clinical biomarker studies recently determined that ONC201 antagonizes the dopamine receptor D2-like subfamily of G protein-coupled receptors (GPCR) [17]. A novel target prediction algorithm called BANDIT analyzed millions of data points, including the molecular structure, *in vitro* efficacy profile, and bioactivity assay results for ONC201. The Bayesian machine-learning approach compared ONC201 with all small molecules with known drug targets and predicted a highly specific interaction with dopamine receptor D2 (DRD2) rather than other members of the family. Experimental GPCR profiling as well as reporter assays confirmed that ONC201 antagonizes the D2-like, but not D1-like, subfamily of dopamine receptors. In addition, ONC201 increased levels of serum prolactin, a clinical hallmark of DRD2 antagonism, in several patients from the first-in-human Phase I study. The potential relevance of this interaction to the antitumor activity of ONC201 is indicated by a range of preclinical reports that have demonstrated anti-tumor efficacy effects of D2-like receptor inhibition in a range of malignancies [18-20]. This molecular interaction is particularly intriguing given that GPCRs are the targets of a large number of marketed drugs for many diseases, with oncology being a striking exception [21].

In summary, ONC201 coordinates a network of anti-cancer signaling effects through an early-stage ISR activation in tumor cells (Figure 2) *via* an apparently unique trigger that leads to pronounced efficacy without harming normal cells. This signaling cascade can explain the previously described late signaling effects of ONC201 such as Akt/ERK/Foxo3a/DR5/TRAIL-dependent apoptosis that has also been reported downstream of ER stress [22]. The profile of ONC201 suggests that the small molecule may engage a novel binding target for oncology.

## PRECLINICAL EFFICACY PROFILE

The efficacy of ONC201 has been demonstrated in numerous cancer cell lines and patient samples that are refractory to chemotherapy and targeted therapies. ONC201 has demonstrated single agent anti-tumor effects in over 15 *in vivo* preclinical cancer models that include subcutaneous, orthotopic, and transgenic and other immunocompetent mouse models in a wide range of malignancies with infrequent oral administration (Supplementary Table 1). Based on consistent preclinical evidence, summarized below in part, ONC201 is currently being evaluated in multiple advanced cancer types that exhibit preclinical sensitivity to the drug candidate.

### ONC201 activity in hematological malignancies

One of the early *in vivo* efficacy evaluations of oral ONC201 was in an Eμ-myc transgenic mouse model that spontaneously causes metastatic myc-driven aggressive lymphomas by approximately 9 weeks of age [1]. Weekly oral administration of ONC201 from weeks 9-12 of age prolonged the median survival of these lymphoma-bearing mice by 4 weeks. All mice survived while on therapy and tolerated ONC201 well. A large *in vitro* efficacy screen of more than 1,000 human cancer cell lines indicated that ONC201 had the highest potency in lymphomas and multiple myeloma (MM) (Figure 3), a finding in agreement with ISR activation by ONC201. Parallel independent investigations have revealed that multiple myeloma cells are relatively sensitive to ONC201, with GI50 values in the nano-molar range that are an order of magnitude below other tumor types [23]. In addition, ONC201 induces the TRAIL pathway and caspase-dependent cell death in non-Hodgkin's lymphoma cell lines [24].

ONC201 strongly induces apoptosis in mantle cell lymphoma (MCL) and acute myeloid leukemia (AML) cell lines as well as patient samples. The activity of ONC201 in these hematological malignancies was determined to be p53-independent, as observed in other tumor types, and is equally cytotoxic in tumor cells with complex karyotypes that are associated with a worse clinical prognosis. Importantly, ONC201 induces high levels of specific apoptosis in *ex vivo* MCL patient samples with clinical resistance to standard-of-care therapies, such as ibrutinib. ONC201 was similarly active *in vivo*, ablating engraftment of human AML cells into NSG mice to significantly prolong their survival relative to untreated cohorts [7]. Caspase-dependent apoptosis by ONC201 has been observed in acute lymphoblastic leukemia (ALL) *via* modulation of Bcl-2 and IAP-family proteins [25].

Single agent ONC201 has pronounced pro-apoptotic activity in primary Sézary cells from advanced cutaneous T-cell lymphoma (CTCL) patients compared with normal PBMCs from healthy donors [26]. Interestingly, ONC201 was more effective in CTCL patient samples and cell lines that are relatively more aggressive and refractory, highlighting its potential clinical utility in such settings. In addition to inducing the TRAIL pathway, mechanistic studies revealed that ONC201 inhibited the phosphorylation of Jak and STAT in CTCL cell lines. The Jak/STAT signaling pathway is critical in the survival of CTCL cells [27] and can be inactivated downstream of ISR activation, suggesting that it is a coordinated downstream element of the described mechanism of ONC201 rather than an independent mechanism.

### ONC201 activity against glioblastoma

The first description of the chemical structure of ONC201 in the literature is a German patent filed in 1973. While there is no evidence that the compound was evaluated in any biological assays, the patent speculates its utility in treating central nervous system disorders. In agreement with this speculation, murine studies revealed that ONC201 penetrates the blood-brain-barrier and is active in brain tumor xenograft models [1]. *In vitro* studies have shown that ONC201 inhibits cellular proliferation, induces TRAIL, and causes cell death in numerous glioblastoma cancer cell lines in cell culture models, including those resistant to the standard-of-care chemotherapy temozolomide. Furthermore, ONC201 has demonstrated potent cytotoxicity against cancer cells isolated from a freshly resected glioblastoma tumor that was resistant to temozolomide, bevacizumab, and radiation [1]. *In vitro* studies of ONC201 in glioblastoma cell lines suggest that its efficacy is not reduced by mutations in p53, EGFR, or IDH1/2 genes or MGMT status.

ONC201 was shown to be highly effective in a subcutaneous xenograft of temozolomide-resistant glioblastoma as a single agent administered as a single dose [1]. ONC201 tripled mouse survival when combined with bevacizumab, the anti-VEGF monoclonal antibody that is FDA-approved for the treatment of glioblastoma [1]. In addition to highly significant activity against glioblastoma *in vitro* and in subcutaneous xenografts, a single dose of ONC201 was sufficient to double the survival of mice harboring aggressive intracranial human glioblastoma tumors [1]. Based on the compelling efficacy in refractory glioblastoma models and its ability to traverse the blood brain barrier, the clinical efficacy of ONC201 is being evaluated as a single agent in a phase II clinical trial in bevacizumab-naïve recurrent glioblastoma (NCT02525692). It is worth noting that bortezomib, which also induces ISR, has shown preclinical activity in glioblastoma [28] and, despite its minimal penetration of the blood-brain-barrier [29], demonstrated some signs of clinical activity in this indication that includes two partial responses in a phase I glioblastoma trial [30].

### ONC201 activity against colorectal cancer

The small molecule library screen that led to the discovery of ONC201 was conducted with a human CRC cell line, which is a tumor type that possesses relatively high sensitivity to ONC201 among solid tumors. ONC201 induces TRAIL and subsequent cell death in numerous human CRC cell lines harboring diverse oncogenic mutations, including those that result in resistance to standard-of-care agents in this setting such as p53 and KRAS. In addition to cancer cell lines, ONC201 was highly effective at inducing TRAIL and cell death in fresh CRC specimens. The latter includes complete eradication of cultured cells from a mucinous adenocarcinoma tumor resected from an 85 year-old female patient who was unresponsive to prolonged treatment with the standard-of-care chemotherapy 5-fluorouracil (5-FU) [1].

*In vivo*, ONC201 induces TRAIL and causes potent anti-tumor effects when administered as a single dose in several human CRC subcutaneous xenografts. Extensive validation of the mechanism of action of ONC201 has been conducted in CRC xenografts in mice including correlative assays and genetic studies that demonstrate engagement of the Akt/ERK/Foxo3a/TRAIL/DR5 signaling axis in tumors [1, 31].

### ONC201 activity against cancer stem cells

Cancer stem cells (CSCs) are a rare subpopulation of stem-like tumor cells that have been shown to drive tumor initiation, disease progression, resistance to cancer therapies, disease relapse and metastasis in various tumor types [32, 33]. The efficacy of ONC201 involves targeting both bulk tumor cells and CSCs in hematological as well as solid malignancies. ONC201 has demonstrated strong preclinical anti-CSC efficacy in CRC, AML and glioblastoma models [34, 35].

ONC201 significantly depleted CD133, CD44 and Aldefluor-positive 5-FU-resistant CSCs in CRC cell lines in a manner that was dependent on Akt, Foxo3a, and DR5. Inhibition of colonosphere formation, as well as tumor growth and serial passage of CSC-initiated tumors, has been demonstrated with ONC201 in CRC [31]. In refractory AML patient samples, ONC201 induced apoptosis in leukemia stem/progenitor cells (CD45 dim+/ CD34+/ CD38-) to an extent that was equivalently observed in non-CSCs [7]. ONC201 inhibited cell proliferation of CSC-enriched neurosphere cultures of newly diagnosed and recurrent glioblastoma patient samples [34] and induced apoptosis in stem-like glioma cells [35]. The capability of ONC201 to be equally effective in the CSC subpopulation precludes a common mechanism of resistance and could augment its long-term therapeutic benefits, especially in advanced stage previously treated drug-refractory disease.

## PRECLINICAL SAFETY PROFILE

ONC201 exhibits an exceptional safety profile given its broad and potent anti-cancer activity which results in a wide therapeutic index *in vitro* and *in vivo* [36]. ONC201 does not significantly impact the cell cycle profile or viability of normal human fibroblasts at concentrations that strongly induce cancer cell death. Lack of ONC201-induced cytotoxicity has been demonstrated in normal fibroblasts, bone marrow cells, and stem-progenitor blood cells. Furthermore, ONC201 does not induce markers of genotoxicity in tumor or normal cells [1, 7]. Interestingly, *in vitro* co-culture studies suggest that normal cells can contribute to tumor-specific cytotoxicity *via* a bystander effect through TRAIL signaling [1], as observed with radiation [37-39]. Examining ONC201-induced downstream signaling in normal cells revealed that DR5 induction is absent, perhaps due to blunted ISR activation, but that a modest amount of TRAIL is induced on the surface of normal and stromal fibroblasts [1, 2].

While *in vitro* studies indicate that ONC201 spares normal cells from cytotoxic effects, the *in vivo* safety profile is also favorable. Dose titration studies in a CRC xenograft model revealed that ONC201 is maximally efficacious at 25 mg/kg, and is equivalently effective with oral or intra-peritoneal administration. Several studies utilized a dose of up to 100 mg/kg without incidence of ONC201-related mortality or overt signs of toxicity. Body weight measurements and histology studies in multiple strains of mice have revealed no evidence of adverse effects of ONC201 administration on normal mouse tissues at effective doses [1, 2, 31].

To confirm the safety profile of ONC201 and enable its translation into the clinic, GLP toxicology studies were conducted in Sprague Dawley rats and beagle dogs with oral ONC201 [36]. The only adverse findings observed in both rats and dogs occurred at the highest doses tested (225 mg/kg in rats and 120 mg/kg in dogs): mild and reversible decreased activity, decreased food consumption, and increased salivation. Interestingly, the mild adverse effects of ONC201 at exaggerated doses in animal studies appeared to PK-related (C_max_), unlike its anti-tumor activity that appears to saturate beyond a given dose level and frequency. The no-observed-adverse-event-level (NOAEL), which is commonly used to determine starting doses in human clinical trials, was at least 42 mg/kg in dogs and at least 125 mg/kg in rats. Both of these NOAELs are approximately 1.25 g in humans assuming standard allometric conversion and a 60 kg body weight for humans [36]. These NOAELs correspond to a dose that is 10-fold above the murine dose of 25 mg/kg that showed anti-cancer efficacy in human tumor xenograft studies.

## COMBINATION THERAPY

**Figure 4:**
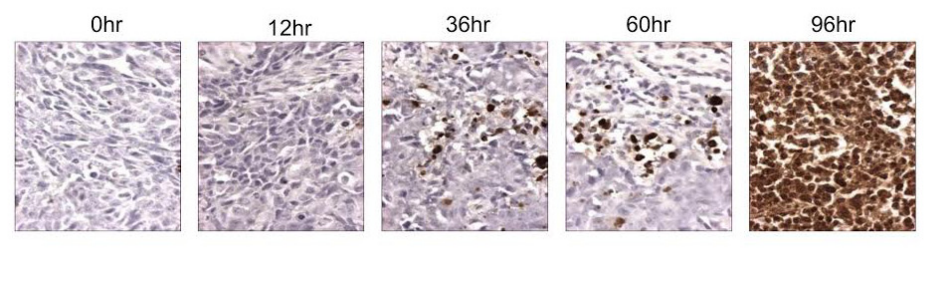
The pro-apopototic activity of ONC201 is sustained TUNEL assay on HCT116 subcutaneous xenografts harvested following a single dose of ONC201 (25 mg/kg) at the indicated time points.

**Figure 5:**
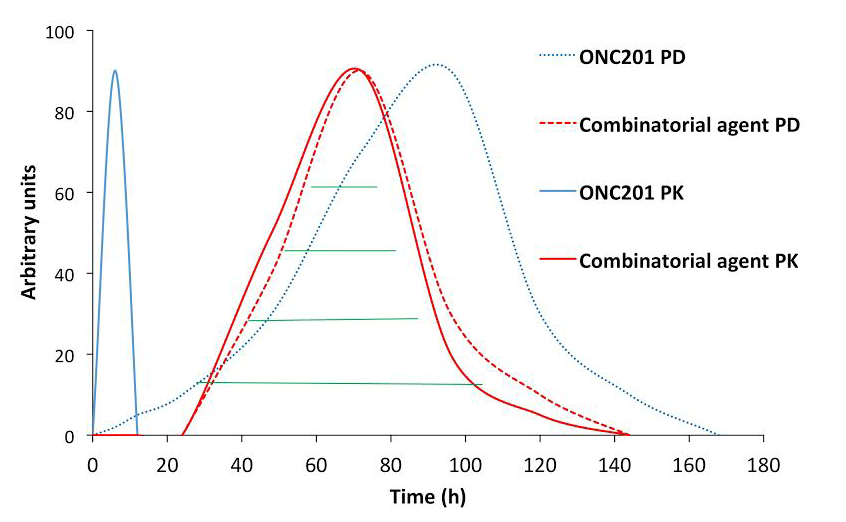
Proposed model for PK/PD-guided administration of ONC201 as a combinatorial anti-cancer agent ONC201 is particularly attractive for combinatorial regimens due to a short half-life and PD that persists for days to weeks. Due to this unique PK/PD relationship, ONC201 could be administered one to two days prior to another anti-cancer agent that would allow for a synergistic PD overlap (green lines) while avoiding drug-drug interactions.

ONC201 has a half-life of about 6-11 hours across different species and PD typically engaged several hours after single-dose administration that persists far beyond its PK (Figure 4). This disconnect between PK and PD is particularly attractive for combinatorial regimens, allowing ONC201 to be administered one to two days prior to another anti-cancer drug that has demonstrated synergistic efficacy in preclinical models. This could allow for a synergistic PD effect without requiring the therapeutics to be systemically present at the same time (Figure 5). Thus, ONC201 may be administered with other therapies using a schedule that significantly diminishes the potential for drug-drug interactions while maintaining cooperative intra-tumoral signaling.

Though ONC201 is effective as a single agent in many cancer models, it is also broadly synergistic with approved and investigational cancer therapies in preclinical models in accordance with its mechanism of action that is distinct and complementary to many other approved cancer therapies. A screen conducted in solid tumor cell lines identified several synergistic interactions between ONC201 and FDA-approved small molecules used in oncology: azacitidine, bortezomib, dacarbazine, hydroxyurea, and sorafenib. In hepatocellular carcinoma, sorafenib combined with ONC201 to synergistically reduce cell viability and cooperatively induce TRAIL, DR5, and cell death through a mechanism that involved IAP and Bcl-2 family proteins. The combination produced an increased rate of complete tumor regression (8/10 tumors) compared to ONC201 (4/10) or sorafenib (0/10) alone following two weeks of therapy in a hepatocellular subcutaneous xenograft while being well tolerated [40].

Synergy between ONC201 and the taxanes paclitaxel and docetaxel has been documented in non-small cell lung cancer cell lines [1]. Single dose ONC201 induced tumor stasis in H460 human non-small cell lung cancer xenografts in athymic nude mice to an extent that was comparable to paclitaxel and superior to docetaxel. The combination of ONC201 with paclitaxel or docetaxel was well tolerated and resulted in complete regressions of > 60% of tumor xenografts [1, 31].

The combination of ONC201 and bevacizumab has also proven cooperative in preclinical glioblastoma and CRC models. In an orthotopic CRC model, this combination reduced primary cecal tumor burden and decreased spread to distal metastatic sites including lung, liver, lymph nodes and peritoneum. The combination of ONC201 with bevacizumab caused no significant changes in body weight. ONC201 was also well tolerated and cooperative in an orthotopic model of glioblastoma where the combination tripled the survival of mice receiving single dose administration [1].

**Figure 6:**
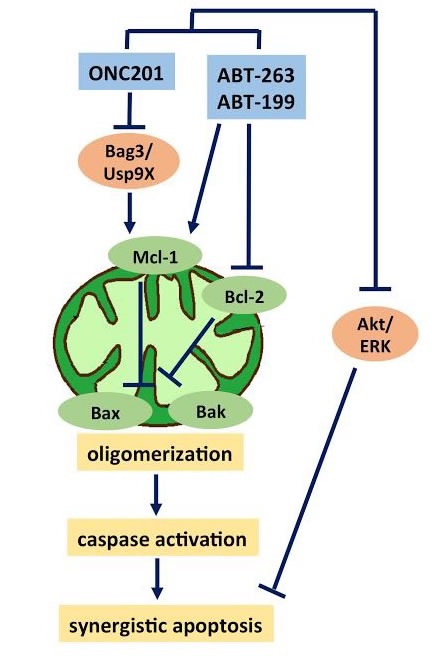
ONC201 synergizes with Bcl-2 inhibitors Bcl-2 overexpression reduces ONC201-mediated cell death while high Mcl-1 levels are linked to ABT-199/ABT-263 resistance. The combined inhibition of Bcl-2 with ABT-263/ABT-199 and Mcl-1 with ONC201 (*via* Bag3/Usp9X inhibition) results in synergistic anti-tumor effects *via* the mitochondrial pathway of apoptosis involving Bax/Bak oligomerization, caspase activation and PARP-cleavage. Improved inhibition of Akt/ERK also contributes to the synergistic effects.

One of the most promising synergistic combinations with ONC201 is with Bcl-2 inhibitors, which has been demonstrated in multiple studies. Mcl-1 knockdown improved the anti-cancer effects of ONC201 in CRC and leukemia cells [7, 40]. Striking synergy was observed *in vitro* with the Bcl-2-specific inhibitor ABT-199 in leukemia cells [7]. Another study reported robust ONC201 synergy with the Bcl-2-family inhibitor ABT-263 in glioblastoma cells *in vitro* and *in vivo* [35]. Bcl-2/Bcl-xl overexpression has been shown to reduce ONC201-mediated cell death and high Mcl-1 levels are linked to ABT-199/ABT-263 resistance. The combined targeting of Bcl-2 and Mcl-1 results in synergistic anti-tumor effects of ONC201 in combination with ABT-263/ABT-199. ONC201-mediated degradation of Mcl-1 occurs *via* down-regulation of chaperone Bag3 and the deubiquitinase Usp9X, both known to stabilize Mcl-1 [7, 35]. Thus, the combination of ONC201 and Bcl-2 inhibitors uses complementary mechanisms of sensitization where each therapy eliminates a key mechanism of resistance for the other agent (Figure 6).

ONC201 single agent and combinatorial efficacy has also been demonstrated in pancreatic cancer [41]. Single agent ONC201 inhibited pancreatic cancer growth *in vitro* and *in vivo via* the Akt/ERK/TRAIL-mediated mechanism. The combination of ONC201 and gemcitabine was well tolerated, inhibited xenograft tumor growth and improved survival in mice [41]. Among hematological malignancies, synergy has also been documented with the combination of ONC201 and cytarabine *in vitro* [24].

Thus, ONC201 combines synergistically and is well tolerated with a multitude of therapies ranging from approved chemotherapies and targeted therapies to investigational agents that can be explored in future clinical trials. Combining ONC201 with immunotherapies is also being evaluated in preclinical studies given its lack of immunosuppression and the ability of the novel agent to stimulate production of TRAIL, which is an effector cytokine expressed by many activated immune cells.

## PHARMACOKINETICS

The PK profile of ONC201 has been determined in mice, rats, dogs, and is currently being determined in humans. ONC201 exposure in beagle dogs following oral gavage dosing at 4.2, 42, and 120 mg/kg ONC201 was dose-dependent and increased with greater ONC201 dose levels (Supplemental Figure 1A) [36]. Exposure to ONC201 was slightly higher in the female dogs relative to the male dogs. The terminal half-life ranged from 4.6 to 7.8 hours in this species.

In rats, exposure to ONC201 was dose-dependent and approximately dose-proportional (Supplemental Figure 1B) [36]. Exposure to ONC201 was slightly greater in female rats after a single oral gavage dose. The terminal half-life ranged from 2.3 to 8.4 hours. The volume of distribution was large, ranging from ~49 to ~103 L/kg in 6 of 8 evaluated profiles, suggesting that ONC201 is widely distributed. The half-life of ONC201 in mice is ~6 hours with intravenous administration as measured by an HPLC-UV assay [1].

The pharmacokinetics of single agent ONC201 in a phase I clinical trial in advanced solid tumors is being evaluated by LC-MS-MS analysis of plasma collected in the first cycle of therapy within 21 days of the first administration of ONC201 [42].

## CLINICAL INTRODUCTION

The clinical safety of ONC201 has been evaluated in a phase I trial [42]. The design was an open-label, dose-escalation phase I trial of mono-agent ONC201 in patients with advanced, refractory solid tumors who had exhausted or refused standard treatment options for their respective indications. The primary objective of this study was to determine the recommended phase II dose (RP2D) of ONC201 administered orally in patients with advanced cancers. Secondary objectives were also assessed including PK and PD. An accelerated dose escalation design, which uses single patient dose cohorts in the absence of significant adverse events, was employed due to the excellent safety profile of ONC201. In accordance with the benign preclinical safety profile of ONC201, the study advanced through five dose levels ranging from 125 mg through 625 mg, with one patient per dose level. Six patients were enrolled at the highest dose level of 625 mg. The study identified a recommended Phase II dose of 625mg with robust safety and PK profiles, ONC201 plasma concentrations in the therapeutic range, preliminary evidence of tumor engagement and prolonged PD. Weekly dosing is under investigation in view of the clinical safety and PK profiles of ONC201 that are expected to permit more frequent dosing without compromising safety.

Several additional clinical trials are now evaluating the clinical activity of ONC201 in multiple tumor types as a single agent (Table 1). The tumor types selected for phase II clinical trials are based on compelling preclinical evidence of efficacy. Given the partial overlap in mechanism with ONC201, it is encouraging that bortezomib has shown signs of clinical activity in several of these indications, including glioblastoma [30], hormone-refractory prostate cancer [43], CTCL [44], non-Hodgkin's lymphoma [45], and multiple myeloma [46]. Several clinical trials are also exploring alternative dosing regimens with ONC201 in the phase I setting.

**Table 1:** Clinical trials with ONC201

Identifier	Phase	Indication	Design	Primary Endpoint	Institution	Status
NCT02250781; NCT02324621	I	Solid Tumors	Accelerated titration; Expansion phase	RP2D	Cancer Institute of New Jersey	Complete; Enrolling
NCT02392572	I/II	Acute Myeloid Leukemia, Acute Leukemia, Myelodysplatic Syndrome	Accelerated titration; Efficacy	RP2D/ORR	MD Anderson Cancer Center	Enrolling
NCT02420795	I/II	Non-Hodgkin's Lymphoma	3 + 3 dose escalation; Efficacy	RP2D/ORR	MD Anderson Cancer Center	Enrolling
NCT02609230	I	Solid Tumors	Dose escalation	RP2D	Fox Chase Cancer Center	Enrolling
NCT02863991	II	Multiple Myeloma	Efficacy	ORR	Fox Chase Cancer Center	Enrolling
NCT02525692	II	Glioblastoma	Efficacy	PFS6	Massachusetts General Hospital/ Dana Farber Cancer Institute	Enrolling

## CHEMICAL PROFILE AND EXTENDING THE THERAPEUTIC POTENTIAL OF ONC201

The pharmacophore of ONC201 is an imidazo [1,2-a]pyrido [4,3-d]pyrimidine heterocycle [47] that offers a unique core structure. The small molecule has several chemical properties that are favorable as a drug candidate: no stereoisomers, highly stable, facile synthesis, adequate aqueous solubility, passively penetrates the blood-brain barrier. A proprietary salt formulation has been created for preclinical and clinical studies of ONC201 that improves the stability and solubility of the compound.

Given the novel pharmacophore of ONC201, its unique mechanism, and its profile that has translated well to the clinic, a medicinal chemistry effort was undertaken to alter drug characteristics such as potency, metabolism, and biodistribution. Structure-function studies identified an inactive linear isomer of ONC201 that does not affect Akt/ERK/Foxo3 signaling, and is unable to induce TRAIL and cell death in tumor cells. This inactive isomer serves as an ideal negative control for preclinical studies [47]. One example where ONC201 analogs may be particularly attractive for development are tumor types where the potency required to achieve efficacy cannot be physiologically achieved by ONC201. A few lead candidates have emerged that exhibit a 10- to 200-fold potency improvements in human cancer cells in a range of tumor types. Thus, ONC201 is the founding member of a novel class of therapies, which are collectively called imipridones based on their core chemical structure, that can extend its attractive therapeutic profile to an array of distinct chemical entities and clinical utilities.

## CONCLUSIONS

ONC201 is a first-in-class, orally active, non-toxic anti-tumor agent currently being evaluated in advance cancer clinical trials. The anti-tumor effects of ONC201 are mediated by an early-stage ISR activation in tumor cells *via* a potentially novel target(s) for oncology that leads to pronounced anti-cancer efficacy at doses without observable toxicity towards normal cells. ONC201-mediated ISR activation involves phosphorylation of eIF2α, which leads to induction of ATF-4 and CHOP. ISR activation leads to ONC201-mediated dual inhibition of Akt/ERK and activation of Foxo3a, which ultimately results in induction of DR5 and TRAIL causing cancer cell death. The robust anti-tumor efficacy of ONC201 has been demonstrated in numerous solid tumors and hematological malignancies in preclinical models as a single agent and in combination regimens. The benign safety profile of ONC201 has been established in a range of animals and is being confirmed in phase I clinical trials. The unique pharmacophore of ONC201 has been extended to create the imipridone class of compounds that possess distinct properties, such as improved potency in tumor types that are less sensitive to ONC201. The atypical discovery and development approach with ONC201 exemplifies how phenotypic drug discovery and parallel, rather than sequential research and development efforts enable synergistic multi-disciplinary information in real-time that can aggregately unveil revelations that might not have been apparent with traditional approaches.

## SUPPLEMENTARY MATERIALS TABLE AND FIGURE


