# Correction: Discovery and clinical introduction of first-in-class imipridone ONC201

**DOI:** 10.18632/oncotarget.28012

**Published:** 2021-10-12

**Authors:** Joshua E. Allen, C. Leah B. Kline, Varun V. Prabhu, Jessica Wagner, Jo Ishizawa, Neel Madhukar, Avital Lev, Marie Baumeister, Lanlan Zhou, Amriti Lulla, Martin Stogniew, Lee Schalop, Cyril Benes, Howard L. Kaufman, Richard S. Pottorf, B. Rao Nallaganchu, Gary L. Olson, Fahd Al-Mulla, Madeleine Duvic, Gen Sheng Wu, David T. Dicker, Mala K. Talekar, Bora Lim, Olivier Elemento, Wolfgang Oster, Joseph Bertino, Keith Flaherty, Michael L. Wang, Gautam Borthakur, Michael Andreeff, Mark Stein, Wafik S. El-Deiry

**Affiliations:** ^1^ Oncoceutics, Inc., Philadelphia, PA, USA; ^2^ Fox Chase Cancer Center, Philadelphia, PA, USA; ^3^ University of Texas MD Anderson Cancer Center, Houston, TX, USA; ^4^ Weill Cornell Medicine, New York, NY, USA; ^5^ Massachusetts General Hospital, Boston, MA, USA; ^6^ Harvard Medical School, Boston, MA, USA; ^7^ Rutgers Cancer Institute of New Jersey, New Brunswick, NJ, USA; ^8^ Provid Pharmaceuticals, Monmouth Junction, NJ, USA; ^9^ Kuwait University Medical School, Kuwait; ^10^ Karmanos Cancer Institute, Detroit, MI, USA; ^11^ The Children’s Hospital of Philadelphia, Philadelphia, PA, USA

**This article has been corrected:** The COI statement has been updated. Please see the revised text below:

## CONFLICTS OF INTEREST

W.S.E-D. is a co-founder and shareholder of Oncoceutics, Inc. W.S.E-D. is fully compliant with institutional disclosure requirements and conflict of interest rules. Dr. Michael Andreeff is also a shareholder of Oncoceutics, Inc. Some of the additional authors are employees or shareholders at Oncoceutics, Inc.

Original article: Oncotarget. 2016; 7:74380–74392. 74380-74392. https://doi.org/10.18632/oncotarget.11814

