# Supplementary material for: Discovery and clinical introduction of first-in-class imipridone ONC201
Source: Oncotarget. 2016 Sep 1;7(45):74380–92. doi: 10.18632/oncotarget.11814 (PMC5342060; doi:10.18632/oncotarget.11814)

Discovery and clinical introduction of first-in-class imipridone ONC201

Supplementary Material

Supplementary Table 1. Summary of ONC201 *in vivo* anti-cancer efficacy studies.

Note: s.c.: subcutaneous, IP: intraperitoneal, PO: oral, IV: intravenous, NA: not applicable, CSC: cancer stem cell

Tumor type	Mouse model	ONC201			Comparison	Combination	CSC-efficacy	Reference
		Dose, Route	Frequency	Efficacy				
Lymphoma	Eμ-myc transgenic	25 mg/kg, PO	1x/week	Prolonged survival	NA	NA	NA	1
Acute Myeloid Leukemia	Patient-derived cells serially passaged in NSG mice (i.v.)	5 μM, *in vitro*	48hrs in vitro	Prolonged survival	NA	NA	Yes	7
Glioblastoma	T98G s.c.	30 mg/kg, PO	single dose	Partial regression	slightly better than bevacizumab	NA	NA	1
	SF767 intracranial	25 mg/kg , PO	single dose	Prolonged survival	survival better than bevacizumab	bevacizumab+ONC201 further improved survival	NA	1
	MGPP-3 s.c.	25 mg/kg, IP	3x/week	Partial regression followed by progression	ABT263 response similar to ONC201	ABT263+ONC201 tumor regression	Yes	30
						better than single agent		
Colorectal	SW480/RKO/HCT116 p53 null s.c.	30/50/100 mg/kg, IP and PO	single dose	Stasis	NA	NA	NA	1
	HCT116 wt/p53 null and DLD-1 s.c.	12.5/25/50/80/100 mg/kg	single dose, 1-2 doses/week	Partial regression	similar or slightly better than rhTRAIL	NA	NA	1, 2
		IP/IV/PO	1x/2-3weeks					
	HCT116 p53 null, intracecal	25 mg/kg, PO	1x/week	slightly reduced primary tumor	better than bevacizumab	bevacizumab+ONC201 improved primary tumor reduction	NA	1
				and decreased metastasis		and decreased lung metastasis		
	DLD1 Aldefluor+ CSCs	50 mg/kg IP	1x/week	Partial regression, reduction of passage	superior to 5-Fluorouracil	NA	Yes	26
	s.c., passage, limiting dilution			decreased tumor initiation and CSC markers				
Head and Neck	Fadu squamous cell, s.c.	50 mg/kg, PO	2x/week	Partial regression followed by progression	radiation resistant	ONC201+radiation slightly better than single agent	NA	
Breast	MDA-MB-231 (triple-negative)	50/100 mg/kg IP	single dose	Partial regression	superior to rhTRAIL	NA	NA	1
Lung	H460 (non-small cell)	30 mg/kg, IP	single dose	Partial regression	superior to docetaxel	complete regressions in combination	NA	1
						with paclitaxel/docetaxel	NA	
Hepatocellular	HepG2	25 mg/kg, PO	1x/week	Partial regression	superior to Sorafenib	complete regressions with ONC201+sorafenib	NA	35
Pancreatic	Panc-1 s.c.	30 mg/kg, PO	daily	Partial regression and prolonged survival	superior tumor regression compared to gemcitabine	ONC201+gemcitabine improved tumor regression and survival	NA	41

Supplemental Figure 1. Pharmacokinetics of single dose ONC201 in (A) Sprague Dawley rats and (B) beagle dogs.

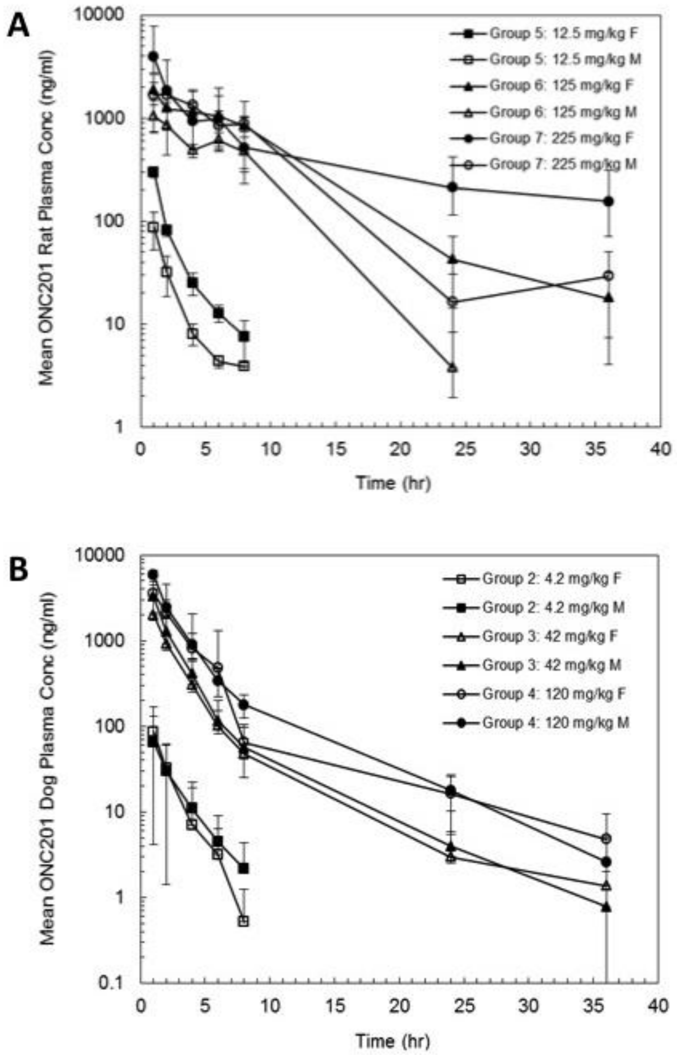

Supplement: Supplementary file 1 [file oncotarget-07-74380-s001.pdf]
